# A conceptual model for healthcare-seeking research and interventions in cutaneous leishmaniasis

**DOI:** 10.1186/s12939-026-02763-9

**Published:** 2026-01-28

**Authors:** Sonali Dinushika Gunasekara, Nuwan Darshana Wickramasinghe, Kosala Gayan Weerakoon, Manoj Sanjeewa Fernando, Chandani Liyanage, Helen Philippa Price, Suneth Buddhika Agampodi, Thilini Chanchala Agampodi, Lisa Dikomitis

**Affiliations:** 1 Department of Community Medicine, Faculty of Medicine and Allied Sciences, Rajarata University of Sri Lanka, Saliyapura, Anuradhapura, Sri Lanka; 2 Department of Health Promotion, Faculty of Applied Sciences, Rajarata University of Sri Lanka, Mihintale, Anuradhapura, Sri Lanka; 3 Department of Parasitology, Faculty of Medicine and Allied Sciences, Rajarata University of Sri Lanka, Saliyapura, Anuradhapura, Sri Lanka; 4 Department of Sociology, Faculty of Arts, University of Colombo, Colombo, Sri Lanka; 5 School of Life Sciences, Keele University, Newcastle-Under-Lyme, Staffordshire, United Kingdom; 6 International Vaccine Institute, Seoul, Republic of Korea; 7 Section of Infectious Diseases, Department of Internal Medicine, School of Medicine, Yale University, New Haven, Connecticut United States of America; 8 Warwick Medical School, University of Warwick, Coventry, United Kingdom

**Keywords:** Healthcare-seeking, Leishmaniasis, Social determinants of health, Model

## Abstract

**Background:**

Healthcare-seeking is not merely a biomedical response to illness but a socially and culturally embedded practice reflecting the dynamic interplay of multiple factors. Traditional health behavioural models often overlook the nuanced socio-cultural, environmental, and systemic dimensions associated with neglected tropical diseases. We developed a conceptual model for healthcare-seeking grounded in the lived experiences of rural Sri Lankan communities with cutaneous leishmaniasis (CL).

**Methods:**

We employed a systematic, participatory, and iterative approach to develop the conceptual model, beginning with stakeholder identification. The problem context was explored using empirical data collected through multimethod studies and expert consultations, which informed the model’s objectives and scope. Data were analysed and synthesised to conceptualise key components and influencing factors of healthcare-seeking in CL. The model was iteratively refined through expert review, with sustained stakeholder engagement ensuring contextual relevance and applicability.

**Results:**

Our conceptual model outlines the healthcare-seeking process for CL, integrating two complementary dimensions. On the horizontal axis of the model, we describe the pathway dimension, consisting of four key landmarks: (1) symptom recognition, (2) perceived health threats, (3) decisions on taking actions, and (4) help seeking from the biomedical sector. The vertical axis shows the determinant dimension, which emphasises both proximal and distal factors that shape healthcare-seeking and is grouped into four aspects: (1) individual factors, (2) disease characteristics, (3) social context, and (4) structural determinants. Some *Individual factors* (i.e. disease awareness, perceived severity) affected the entire pathway, whereas others (i.e. perceived treatability, psychosocial impact, and costs) influenced decisions on when, where and how to seek healthcare. *Disease-related characteristics*, including clinical manifestations, prevalence, transmission, and progression, played a critical role, while factors within the *social context*, such as family and neighbourhood cohesion, health beliefs, and myths about CL, further shaped healthcare decisions. Broader *structural determinants*, including health and non-health policies, health literacy, media influence, medical pluralism, and healthcare system factors, indirectly but significantly affected the entire process.

**Conclusions:**

This conceptual model presents the dynamic interplay of cognitive, social, cultural, and structural factors shaping healthcare-seeking in CL. By mapping how individuals navigate illness pathways within broader societal and systemic contexts, this model provides a holistic understanding, informing the design of interventions to improve healthcare-seeking in CL at different levels.

## Background

Cutaneous leishmaniasis (CL) is a neglected tropical disease (NTD) endemic in 90 countries, including Sri Lanka [1]. CL is transmitted through infected sandflies and manifests as chronic, slowly healing skin lesions that may appear as nodules, ulcers, or plaques, often leaving disfiguring scars [2]. CL leads to considerable physical, psychological, and social consequences, particularly due to visible scarring and the requirement of painful and long-term treatment [3, 4]. The control of the disease is hindered by limited prevention measures and multiple barriers to timely healthcare access [3, 5]. Early healthcare-seeking in CL is a challenge due to poor disease awareness [6–9], misconceptions [10, 11] and health system accessibility-related issues [12, 13]. Current treatment options rely mainly on antimonial drugs (intralesional or intramuscular), which are effective but require multiple clinical visits and may cause adverse effects [5]. Prevention largely depends on vector control, personal protective measures, and community awareness improvement [14]. In response to persistent challenges, the WHO Roadmap for NTDs 2021–2030 emphasises integrated and equitable strategies for controlling and eliminating these conditions, with a particular focus on skin-related NTDs like CL [15]. However, provision of integrated and equitable services demands a comprehensive understanding of social, economic, cultural, and environmental determinants that ultimately affect health behaviours [16, 17]. Over the past century, numerous scholars and researchers have advanced the understanding of health and illness behaviours, contributing to the development of various evolving models, though limitations remain in comprehensively understanding healthcare-seeking patterns in CL.

Health-seeking behaviour is a multidimensional concept that has evolved across disciplines, including sociology, anthropology, psychology, and public health [18]. A widely cited definition describes health-seeking as ‘any activity undertaken by individuals who perceive themselves to have a health problem or to be ill for the purpose of finding an appropriate remedy’ [19]. The evolution of health-seeking reflects a progression from individual to systemic perspectives. The foundation for conceptualising health-seeking was laid by Parsons (1951) in his work on ‘sick role’, which framed illness as a social role with rights and obligations, including the duty to seek competent medical help [20]. Mechanic (1962) expanded this to the concept of ‘illness behaviour’, recognising that symptom perception and health-seeking are shaped by cultural and social learning [21]. Suchman’s (1965) five-stage model [22] and Kasl and Cobb’s (1966) [23] differentiation between health, illness, and sick-role behaviours were milestones in understanding health-seeking as a sequential and socially embedded process. Later, Fabrega (1973) [24] and Chrisman (1977) [25] introduced more elaborative stage models that acknowledged sociocultural interpretations, decision points, and lay networks influencing treatment choices.

Different theoretical models and frameworks have guided research on health-seeking behaviour. These can broadly be categorised as health psychological models, anthropological and cultural models, sociological and systemic models, and multilevel and integrative frameworks. Health psychological models such as the Health Belief Model (HBM) [26] are important for predicting preventive health behaviours, assuming that individuals make rational health decisions based on cognitive determinants such as perceived susceptibility, perceived severity, benefits and barriers, cues to action, and self-efficacy [27]. However, its main focus on individual perceptions limits its ability to capture social and structural influences on health behaviours. Anthropological and cultural models, including those of Fabrega [24], Igun [28], and Zola [29], conceptualise health-seeking as a process influenced by sociocultural beliefs, local interpretations of illness, and the pluralistic nature of healthcare systems. Kleinman’s model of medical systems [30] further recognises the coexistence of popular, folk, and professional sectors, highlighting that people’s pathways to care are culturally mediated. A recent theoretical model by Arnault (2018) describes cultural determinants of health-seeking as the shared beliefs, values, norms, and social expectations within a cultural context that shape how individuals perceive and interpret symptoms, and decide whether, when, and from whom to seek help [27]. Sociological and systemic models, such as Andersen’s behavioural model of healthcare utilisation [31], group determinants into predisposing, enabling, and need factors. Although comprehensive, such models are often criticised for overlooking cultural and structural contexts that shape human health behaviour. Carrillo et al. (2011) describe healthcare access barriers as multifaceted obstacles, such as cultural, linguistic, economic, geographic, and systemic factors, that contribute to persistent disparities in the utilisation and quality of healthcare services [27]. Integrative frameworks, such as Engel’s Biopsychosocial model [32] and the WHO’s Social Determinants of Health (SDH) framework [33], extend these perspectives by recognising the interactions between biological, psychological, social, and structural domains. These frameworks move beyond individual explanations and place health-seeking within a wider system of determinants that influence access, equity, and outcomes. While these frameworks are not explicitly designed to explain healthcare-seeking behaviour, a critical understanding of these models and frameworks, their applications and limitations is particularly significant for NTDs like CL, which disproportionately affect individuals living in poverty and resource-limited settings, and where sociocultural and systemic barriers impact healthcare access [2].

In Sri Lanka, CL is a substantial public health problem, with an increasing disease burden, reporting more than 3000 cases annually [34]. In 2023, the average annual CL incidence rate per 100,000 population in Sri Lanka was nearly 1.5 [35]. During the period 2009–2023, the average annual CL incidence rate per 100,000 population was highest in Hambantota district (68.32), followed by Polonnaruwa (52.21), Anuradhapura (40.30), Matara (26.38), Matale (25.48), and Kurunegala (16.21) districts [35]. Leishmaniasis in Sri Lanka is caused by *L. donovani*, the same parasite that causes VL elsewhere, including in India [36]. However, most of the early CL lesions in Sri Lanka are typically small, slow progressing, and often less complicated if managed early. Yet ulcerative, large and complicated lesions have also been reported [4, 37, 38]. In the global context of NTD control, two main approaches have been widely debated: vertical and horizontal strategies [39]. Vertical approaches involve disease-specific programmes implemented through targeted campaigns or dedicated delivery channels, whereas horizontal approaches integrate NTD-related interventions into existing primary healthcare and community systems [39]. In Sri Lanka, CL control follows a more horizontal model, where case detection, diagnosis, and treatment are embedded within the routine public healthcare system under the technical guidance of the Anti-Malaria Campaign [35], which functions as the national focal point for leishmaniasis control. The current study is part of the ECLIPSE research programme, Empowering people with Cutaneous Leishmaniasis: Intervention Programme to improve the patient journey and reduce Stigma via community Education. This is an applied health research programme, with research teams in Brazil, Ethiopia, Sri Lanka and the United Kingdom [40]. The main characteristic of ECLIPSE is its interdisciplinary approach, bringing together experts in anthropology, sociology, parasitology, public health and health communication. Crucially, ECLIPSE is underpinned by robust community engagement and involvement (CEI) [40, 41]. Through extensive years-long fieldwork, we explored the nature of community disease awareness [42], local interpretations and health-related behaviours associated with CL before seeking healthcare [43], patient journey [44], as well as the psychosocial burden [4] and the stigma associated with CL [45] in rural Sri Lanka. Our findings reveal the complex and interdependent influences of individual, social, and structural factors on healthcare-seeking behaviour, highlighting the need for a more comprehensive and contextually grounded framework. Given the limitations of existing frameworks, we developed a bespoke conceptual model to inform evidence-based policies and interventions aimed at improving early healthcare-seeking in CL. This conceptual model integrates the healthcare-seeking pathway and the key influencing determinants.

## Methods

### Study setting

All empirical work was conducted in the Anuradhapura District, Sri Lanka, which has recorded some of the highest incidence rates and prevalence of CL in Sri Lanka over the past decade [36]. By the end of 2020, at the commencement of ECLIPSE empirical data collection, a total of 344 cases of CL had been reported in the district [46]. For the qualitative data collection, we selected three Divisional Secretariat (DS) areas (third-level administrative divisions of the country) within the Anuradhapura District based on recorded CL incidence in 2020. Anuradhapura is the largest district in Sri Lanka, with a land area of 7179 km^2^, located in the dry zone, where the majority of people engage in agricultural activities. The district has a total population of 929,540 (2019) and comprises 2584 villages, 694 Grama Niladhari Divisions, and 22 DS divisions [47]. More than 90% of the population in the district resides in rural areas. Several studies have found that the Anuradhapura District exhibits favourable environmental and climatic conditions that create an optimal habitat for the proliferation of *Phlebotomus* spp., the primary insect vector for leishmaniasis in Sri Lanka [48, 49].

Primary healthcare services in Sri Lanka are delivered through the public and private sectors. The public sector offers a free and universal healthcare system. Within Sri Lanka’s healthcare system, biomedical (Western) and traditional medical systems encompassing Ayurveda, Siddha, Unani, and *Deshiya Chikitsa* operate in parallel. While all these systems are officially recognised, the biomedical system remains the predominant mode of healthcare delivery in Sri Lanka [50]. In the Anuradhapura District, the public healthcare sector comprises a hierarchical network of facilities, including one Teaching Hospital providing tertiary care, six Base Hospitals offering secondary care, 33 Divisional Hospitals and 21 Primary Medical Care Units (PMCUs) delivering primary care services [51]. The private sector encompasses 257 biomedical healthcare facilities [51]. Additionally, the district hosts 25 state-run Ayurveda hospitals with outpatient services and Medical Officer of Health (MOH) centres supporting preventive health services [51]. Among these facilities, CL treatment services are only available at the Teaching Hospital and two Base Hospitals across the district. All three ECLIPSE field sites are located less than 50 km away from a public sector hospital with CL treatment services.

### Model development

**Fig. 1:**
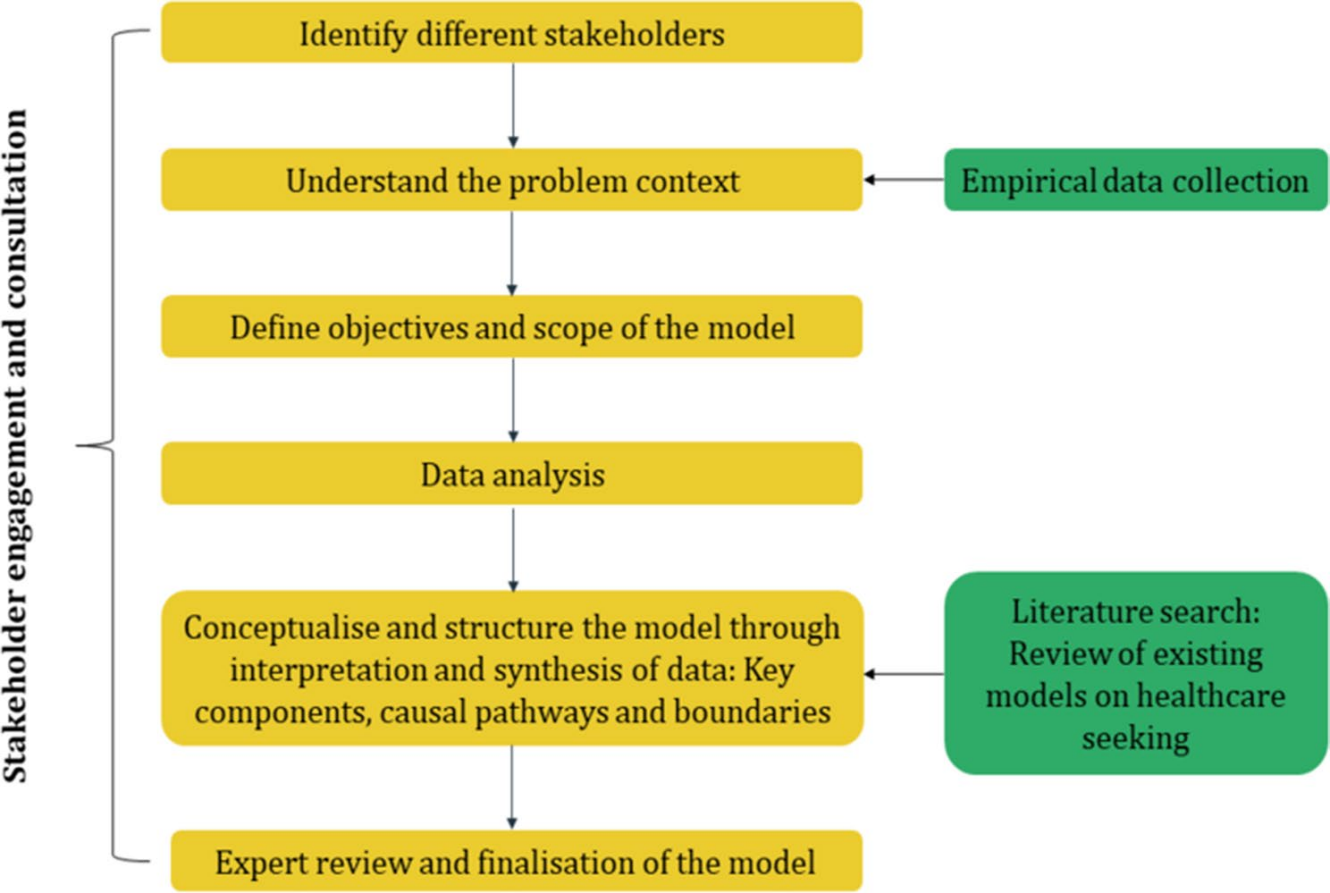
Process of conceptual model development

We developed a novel conceptual model on healthcare-seeking in CL using a systematic, participatory, and iterative six-stage process, guided by the approaches of Naeem et al. (2023) [52] and Squires et al. (2016) [53]. We used an engaged approach at all phases of model development to ensure its relevance, context validity, and public health utility. The stakeholder inputs guided the early conceptualisation of the model, methodological decisions, and results interpretation and validation, facilitating an iterative and participatory process grounded in both professional practice and the lived experience of people with CL. We explain each stage of the conceptual model development process in detail, following Fig. 1.

### Stage 1: stakeholder identification

As part of the broader ECLIPSE programme, we strategically identified the relevant stakeholders, whose knowledge, experiences, and perspectives were significant for understanding the context of the disease and affected communities. Two stakeholder groups were established: a regional-level Community of Practice (CoP), comprising professionals from health, education, agriculture, media, rural development and administration; and Community Advisory Groups (CAGs), consisting of individuals with lived experience of CL, community gatekeepers, and other community members [54]. Apart from these, our research team included the subject experts in public health, parasitology, anthropology, health promotion, and sociology. While these stakeholders were initially engaged to support the overarching goals of the ECLIPSE programme, their insights were also instrumental in informing the development of the conceptual model. The CoP group was engaged to provide policy-level and intersectoral insights, while the CAGs contributed to understanding the lived experiences and contextual knowledge of CL to ground the model in community realities. Subject experts were engaged to fulfil distinct yet complementary roles: public health and health promotion experts to ensure the relevance and public health use of the model; parasitologists to understand clinical and biological aspects of CL; anthropologists and sociologists to guide the methods of empirical data collection.

### Stage 2: understanding the problem context

**Fig. 2:**
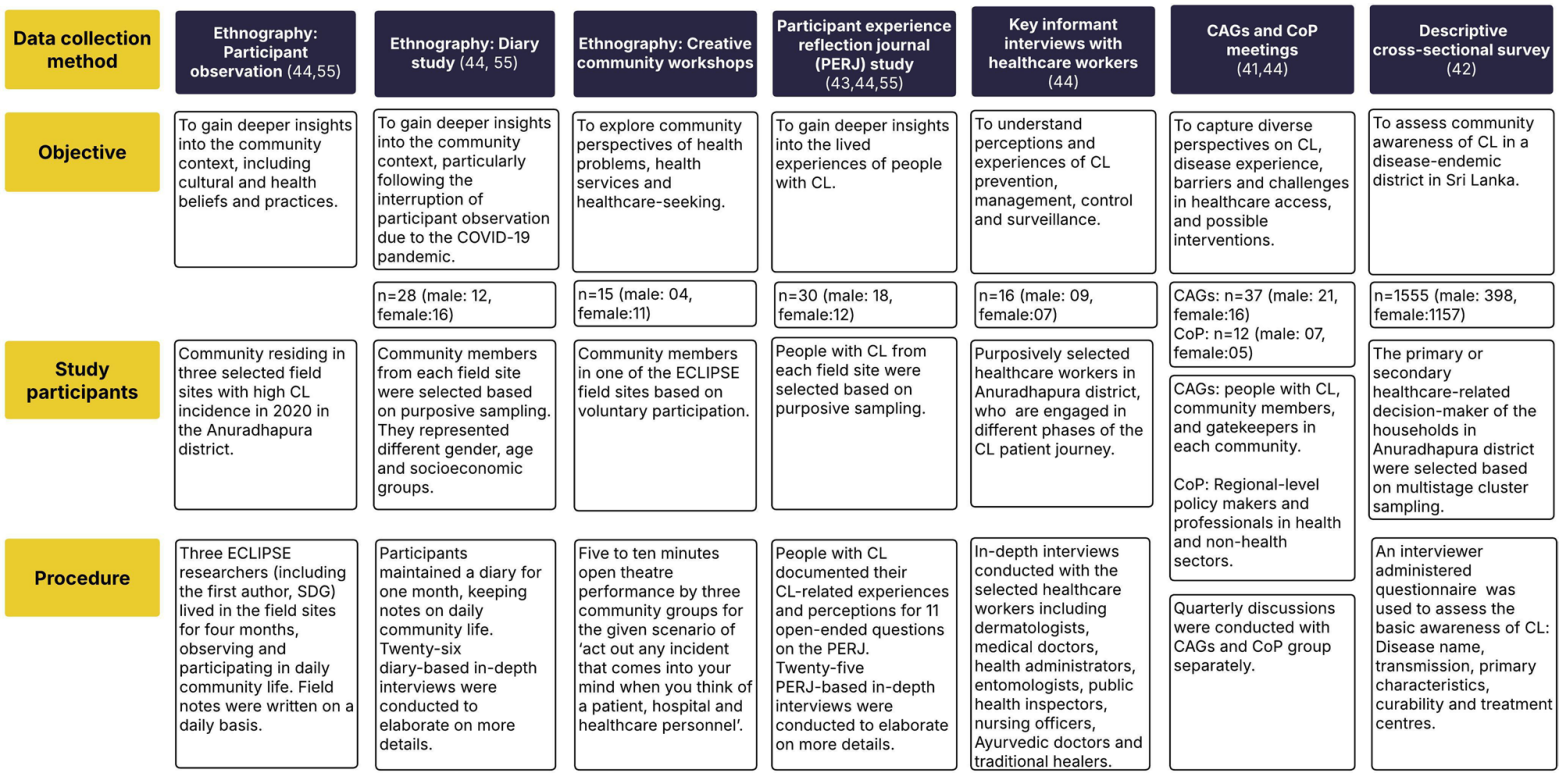
Data sources used in the development of the conceptual model on healthcare-seeking behaviour for CL. This figure depicts the data sources within the ECLIPSE programme that collectively informed the development of the conceptual model. Each data collection method was designed to build upon the insights of the preceding ones, where early ethnographic and participatory approaches guided the development of subsequent data collection tools such as PERJs, interview guides and the survey. The integration of these complementary data sources ensured that the model was empirically grounded, contextually relevant, and reflective of the realities of affected communities and the broader health system

Empirical data collection within the ECLIPSE programme was carried out between January 2021 and February 2023, employing a multimethod qualitative study as reported in our previous publications (43,55) including participant observation, auto-ethnographic diary study, Participant Experience Reflection Journals (PERJ), interviews and a Creative Community Workshop (CCW) [55] to understand perceptions and lived experiences of CL, alongside a district-wide descriptive cross-sectional survey assessing community awareness of CL [42]. We also conducted a review of systemic gaps in the leishmaniasis surveillance system in Sri Lanka [56]. We held frequent discussions with stakeholders on the findings of these studies, discussing the reasons and implications at different levels of society, health and non-health systems. Integrating insights from these diverse data sources was instrumental in developing and rooting the conceptual model within real-world contexts. Further details on the data collection methods, objectives, study participants, and procedures which informed the development of the model are provided in Fig. 2.

### Stage 3: defining objectives and scope of the model

We defined the objectives and scope of the conceptual model via a series of iterative discussions in collaboration with the subject experts, drawing on inputs from other stakeholders. Public health and health promotion experts emphasised that the model should reflect the public health utility in improving healthcare-seeking in CL. Social scientists and anthropologists involved in the process underscored the need to represent all three sectors of the health system described by Kleinman [30], the popular (i.e., people with CL and affected community members), folk (i.e., traditional healers), and professional sectors (i.e., health professionals including medical doctors) to ensure the model adequately reflects the complexity of healthcare-seeking. Complementing these perspectives, parasitologists and dermatologists facilitated understanding of how biological and clinical manifestations of CL influence healthcare-seeking and how they can be incorporated into the model. Through interdisciplinary consensus, we determined that the model’s core constructs should remain adaptable to different contexts and diseases, allowing for broader applicability in diverse healthcare settings.

### Stage 4: data analysis

We employed a combined approach, amalgamating data from already published ECLIPSE study components [4, 42, 43, 56] and analysis of new data sources with a specific focus on healthcare-seeking in CL (Fig. 2). In the process of this analysis, we identified codes and themes based on the objectives and the scope of the model (Stage 3). We integrated all these findings to categorise the proximal (immediate and direct factors) and distal factors (broader and underlying factors) influencing key landmarks of the healthcare-seeking pathway.

### Stage 5: Model conceptualising and structuring

We synthesised our findings into a structured model integrating the pathway and determinants of healthcare-seeking in CL. The pathway was delineated by analysing the sequence of actions and decisions taken by people with CL, while the determinants influencing this process were layered across multiple levels to illustrate their interrelationships and underlying complexity. Throughout this process, we developed the constructs of the conceptual model with a clear focus on their applicability within a public health approach. Additionally, we reviewed the well-established health behaviour models to contextualise our findings within broader theoretical frameworks and enhance the interpretation of data. The constructs identified inductively in our model align with those in established theoretical frameworks, such as HBM [26], SDH framework [33], Kleinman’s model on health systems [30], and Suchman’s model [22] of the healthcare-seeking pathway. Therefore, we have adopted the relevant terminology from these existing frameworks to represent and communicate our constructs.

### Stage 6: expert review and finalisation

We refined and finalised the model through subject experts’ feedback on its face validity, scope, and potential for informing future interventions to improve early healthcare-seeking in CL. While expert consultation was essential to ensure scientific rigour, we acknowledge the inherent power dynamics between researchers, experts, and community stakeholders, and therefore aimed to maintain a participatory and reflexive process that valued all forms of knowledge equally throughout the model development process.

## Results

**Fig. 3:**
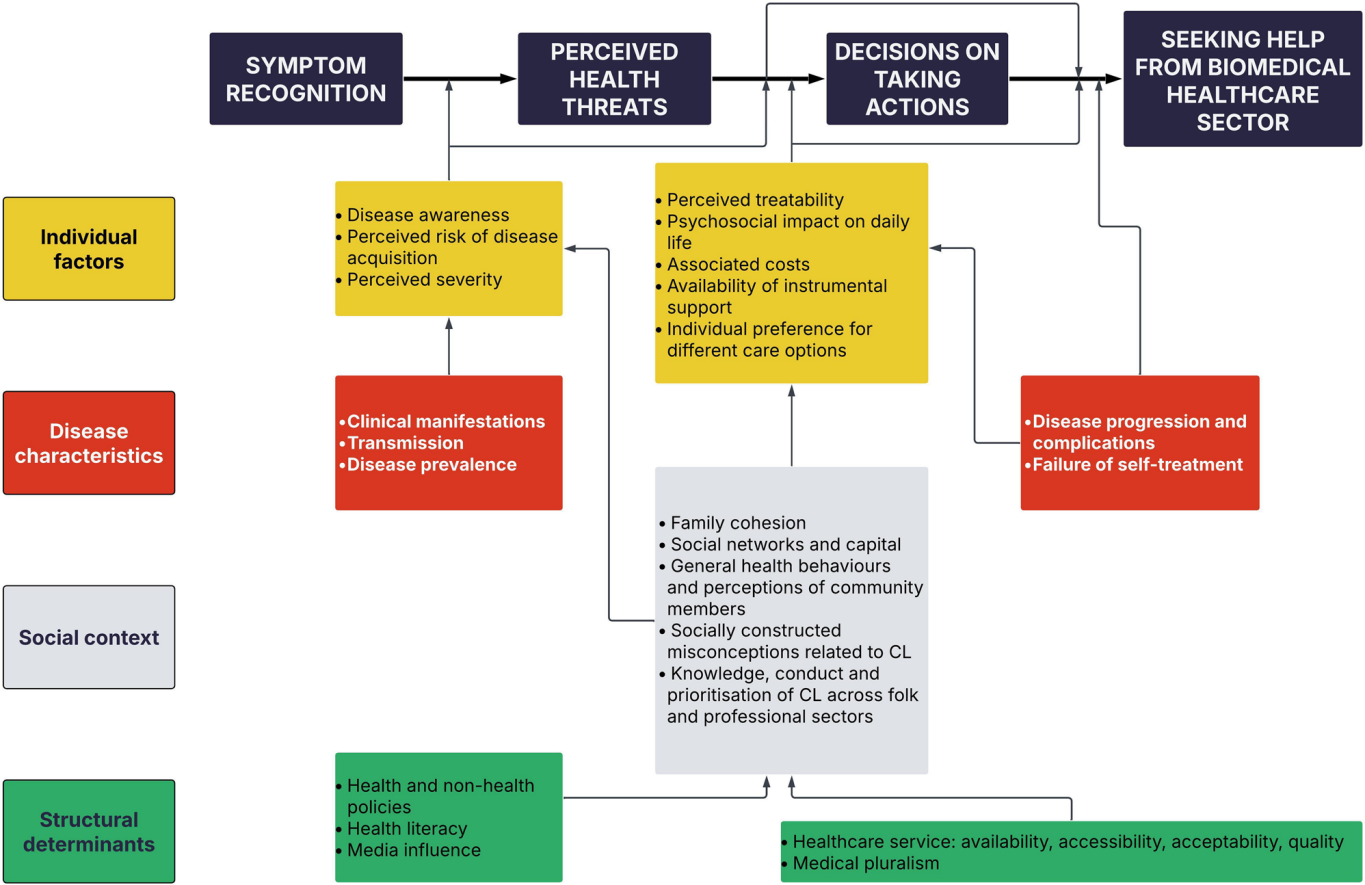
Conceptual model on healthcare-seeking in cutaneous leishmaniasis

Healthcare-seeking in CL is a complex and dynamic process, wherein individuals pass through key stages influenced by various factors. Our conceptual model has two axes and conceptualises the interconnectedness of influencing factors, emphasising their collective impact on healthcare-seeking in CL (Fig. 3). On the horizontal axis of the model, we present four landmarks of the healthcare-seeking pathway [1]: symptom recognition [2], perceived health threats [3], decisions on taking actions, and [4] seeking help from the biomedical healthcare sector. The vertical axis of the model shows the factors of healthcare-seeking grouped into four aspects [1]: individual factors [2], disease characteristics [3], social context, and [4] structural determinants.

### Four landmarks of the CL healthcare-seeking pathway

The healthcare-seeking pathway for CL begins with symptom recognition, as individuals become aware of changes in the skin and developing CL lesions. Subsequently, individuals perceive the CL lesion as a health threat. Individuals then determine whether to act or remain inactive, including where to seek healthcare. The actions can include self-management as well as traditional or Ayurvedic treatment, as we previously reported [43]. Finally, individuals who decide to seek medical care identify and utilise biomedical healthcare facilities, consulting healthcare professionals for diagnosis and treatment.

### Factors affecting healthcare-seeking in CL

We describe the factors of healthcare-seeking in CL and how they manifest in real-life contexts, as reflected in the participants’ ad verbatim excerpts (E1–E27) drawn from different data sources (Table 1). These excerpts, provided in Table 1, serve as supporting evidence for the factors presented in our conceptual model.

**Table 1:** Participants’ excerpts illustrating real-life manifestations of determinants influencing healthcare-seeking for CL

Determinants Landmark	Individual factors	Determinants in the social context	Structural determinants
Symptom recognition and perceived health threats	**Disease awareness** E1: *We had never heard of this disease before. I was wondering why and how such a disease occurs.* (A housewife, excerpt from a participant observation field note) E2: *Since my father had a similar lesion, which was leishmaniasis, I thought it would be best to see a doctor in my case.* (A salesman, PERJ) **Perceived risk of disease acquisition** E3: *There was a wedding ceremony at one of my relatives’ houses, who had leishmaniasis. I spent a few days there helping them and was very tired afterwards. I felt suspicious that I got the disease since I worked in their yard, where sandflies might have been living.* (A male photographer, post-PERJ interview) **Perceived severity** E4: *I feel that this disease is quite serious. The doctors explained that the animal which transmits this disease lays eggs in the wound, and those eggs can travel through the bloodstream to the kidneys and cause damage.* (A retired bank worker, post-PERJ interview) E5: *Some people have big wounds. But I learned that this disease is not contagious from person to person. So, it is not a big issue.* (A housewife, post-PERJ interview)	Popular sector **Social networks and capital** E10: *Upon arriving in the village, our first stop was the petti kade (small roadside shop made with wooden boards). Those boards were covered with various notices, announcements from the death benevolent society, the Farmers’ Society, and the temple committee, turning this spot into a small but vital community information hub. People waiting for the bus exchanged casual conversations at the shop, while others stopped by in the evening for a tea. Gossip flowed freely there. We learned about several people with leishmaniasis in the area at this small shop.* (Excerpt from a participant observation field note) E11: *I did not feel scared the day I saw the wound. But my neighbour had a similar wound and had taken treatment. She looked at mine and said, ‘You have got sandfly disease. You should get treatment for it’.* (An older adult, PERJ) **Socially constructed myths and misconceptions of CL** E12: *Some people’s wounds were very large. Those were so disfigured that it was unpleasant to even look at them. I spoke to one of them. He said, ‘The sandfly lays eggs within the body, and those eggs spread throughout the body with time’.* (A housewife, post-PERJ interviews)	**Impact of health and non-health policies** **Lack of training for healthcare professionals** E18: *According to my knowledge as a medical administrator, more than a thousand cases of CL have been identified during the last year. Capacity building of the health staff, including medical officers and PHLT, is crucial at this moment, however, it remains insufficient.* (A provincial level stakeholder, stakeholder meeting) **Lack of research-driven approaches** E19: *As a clinician, I see a major gap in CL prevention, which could be more cost-effective than treatment. This needs to be research-driven. We know at least two sand fly species transmit the disease, but there’s little research on whether transmission occurs human-to-human or via an animal reservoir. Entomology studies are lacking. In Sri Lanka, CL is caused by Leishmania donovani, which currently only leads to CL. But in India, the same organism causes visceral leishmaniasis. It’s a small genetic shift. What if ours evolves to do the same? (laughs).* (A former dermatologist, an interview) **Lack of evidence-based, updated guidelines for CL prevention** E20: *I would say that there should be some degree of consensus. We cannot find everything at once. With the current evidence, a guideline should be made and implemented. When there is new knowledge, we have to revise the circulars. We have to take all the existing evidence and appoint a committee by the ministry to make some kind of guideline and put it into practice.* (A regional level stakeholder, stakeholder meeting) **Negligence of CL at the policy level during other health challenges** E21: *To tell the truth, surveillance activities are being abandoned. Entomology officers and public health inspectors cannot go to the field investigations due to a lack of fuel within the country. So, the reporting of leishmaniasis case numbers has fallen during the past four weeks. I had to stop leishmaniasis and malaria vector surveillance as there were no vehicles. But dengue surveillance activities were carried out as usual. They issue only 15 litres of fuel per day. We cannot exceed that.* (A regional level stakeholder, stakeholder meeting) **Cultural notions of gender equity** E22: *We, as men, have to accept that women are the strongest. They do take care of the households, support us in farming and actively take the responsibilities in village-level societies.* (A male CAG member, stakeholder meeting) **Media influence** **Social media influence** E23: *When you search on YouTube, you’ll find a lot of information about sand flies and this disease. There are many photographs of leishmaniasis wounds. We usually look at YouTube when there is something new. So, we searched for leishmaniasis as well.* (A mason, post-PERJ interview)
Decisions on taking actions	**Perceived treatability** E6: *It is curable, so the most important thing is to start treatment as soon as you are diagnosed. If left untreated, it can be dangerous.* (A female daily wage labourer, PERJ) **Psychosocial impact on daily lives** E7: *Back then, there were a lot of farming activities going on. So, I just put on some ointments and kept working instead of going to the hospital*. (A farmer, PERJ) E8: *I had to leave home around 5.30 in the morning. My grandson or daughter had to accompany me to the hospital as I was too old to go there on my own. With my age, it was so difficult to wait in the long queue.* (An older adult, PERJ)	**General health behaviours and perceptions of the community** E13: *The main issue is that many people only seek treatment after something happens, or when a doctor recognises the issue. Ignorance leads to a reluctance to attend routine checkups. As an example, by the time doctors diagnose kidney disease, the kidney function is almost lost, leaving less chance of survival.* (A retired school teacher, post diary interview) E14: *It is normal to get cuts and wounds when we do farming activities. It will get better on its own. We usually apply a local ointment called Bilan or else herbal plants. It gets better in a day or a week.* (A farmer, post-PERJ interview)	
Seeking help from the biomedical healthcare sector	**Individual preference over different healthcare options** E9: *In private channel centres, we do not have to wait in line most of the time. Some people think that the medicines given at private centres are good. Others say that they do not get better with the medicine from the government hospital. So, people do not think about expenses for private medical centres.* (A farmer, post-PERJ interview)	**Folk and professional sectors** **Knowledge and conduct of traditional healers** E15: *The sandfly that you talk about is a toxic insect, right? For example, there are wasps, bees and other insects like that. They enter toxins into your body. When the sandfly bites you, you get something in the shape of a flower. It takes a while for it to develop into that, though. Probably it has been at least two years since the last time that I saw those patients. I just need a piece of lime to rub on the wound to remove the toxins there.* (A traditional healer, an interview) **Knowledge and conduct in the professional sector** E16: *I haven’t had many patients with CL in my practice. I’ve seen three CL patients, and one patient came to my house last month. I was able to identify it as leishmaniasis. I know that I am not the best person to treat it, and that it is a Notifiable disease. So, I referred her to the biomedical system, saying that she might have CL.* (An Ayurvedic medical officer, an interview) **Lack of prioritisation of CL within the professional sector** E17: *When a dengue case is reported, we take immediate action. In contrast, leishmaniasis is not given the same urgency. Even within health departments, despite discussions, there is little concern for leishmaniasis.* (A supervising public health inspector, an interview)	**Health service-related factors** **Healthcare service availability** E24: *In Padaviya (one of our ECLIPSE field sites), I think the high disease prevalence and case detection have contributed to the large number of patients we identify. Maybe one reason is that this is a peripheral centre, and only those from that particular village come here. So, mostly early presentations are seen. Therefore, not much diagnostic difficulty occurs*. (A dermatologist, an interview) **Poor physical accessibility** E25: *The public transport service here does not run on time, almost to the point where it feels like there is no transport service at all. The main reason is that there are no proper roads passing through our village. In case of an emergency, there are three-wheelers or vans that people can pay for and use to travel. And during the rainy season, the tank overflows and blocks all the roads. If there were a proper road passing through this village and a regular bus service, accessing hospitals would be much easier.* (A retired school teacher, diary study) **Financial accessibility of health services** E26: *I directly went to the government hospital to check my lesion because they provide all the necessary medicines for every disease, free of cost, which is a great relief for me. How much would it cost if we had to get it done at our own expense?* (A male driver, post-PERJ interview) **Supportive actions to increase accessibility for specific groups** E27: *The leishmaniasis clinic is usually held on Wednesdays, but school children and working individuals with weekday commitments can attend on Saturdays. The staff is very flexible in accommodating patients’ needs.* (A medical doctor, an interview)

Note: Our conceptual model comprises four main categories of determinants influencing healthcare-seeking behaviour: individual factors, disease characteristics, social context, and structural determinants. However, this table presents illustrative excerpts for only three of these categories: individual, social, and structural factors. This decision was made to avoid duplication, as the comprehensive findings and participant excerpts related to disease characteristics, including perceptions of symptoms, disease severity, and transmission beliefs, have been thoroughly explored and published in a separate paper [43]

### Individual factors of CL healthcare-seeking

The healthcare-seeking pathway in CL is influenced by various individual or patient-level factors (Fig. 3). Individual factors such as disease awareness, perceived risk of disease acquisition and perceived severity influence the entire healthcare-seeking pathway. Perceptions of treatability, the psychosocial impact of the disease on daily lives, financial constraints, support availability, and personal healthcare choices become particularly salient in the latter phases, directly informing the decision-making process.

### Disease awareness, perceived risk of disease acquisition and severity

During our ethnographic study, we found that CL was largely unfamiliar within communities, with most participants unaware of the disease before their own experience (E1). This was supported by our cross-sectional survey, which showed a low level of basic awareness of CL among the community (3.6% of 1555 participants), including limited awareness of the disease name, insect vector, symptoms, and treatment [42]. Individuals with prior exposure, either through personal illness or caregiving or close contact with someone affected by CL, were more likely to recognise symptoms early and seek biomedical care (E2).

The perceived risk of acquiring CL influenced how people interpreted the initial lesion, making them more likely to identify it as CL. People described certain environments, such as manna grass, banana bushes, uncleared forest areas, unplastered walls, and decaying leaves, are conducive to sandfly habitats. Our survey findings indicated that individuals with some knowledge of CL had a higher perceived risk of acquiring the disease [42]. When individuals developed a lesion and had prior knowledge of CL, including its symptoms and sandfly habitats, they were more likely to suspect the lesion as CL with recent exposure to such environments (E3). The perceived risk of acquiring CL was high when they concurrently received information about CL and its transmission through media sources such as newspapers or television news, reinforcing their suspicion and facilitating symptom recognition. Having family members or neighbours with CL, along with increasing CL cases in their communities, further increased individuals’ perceived risk of acquiring the disease.

People who had heard of CL often exhibited higher perceived severity. Those who suspected the lesion to be CL or something ‘unusual’, and those with large or facial lesions, particularly young female adults, were more likely to view it as a health threat and seek early biomedical care. Some perceived CL as ‘dangerous’, ‘severe’ or ‘burdensome’ due to misconceptions and others’ experiences (E4). Perceived severity was increased by factors such as the high number of required injections during CL treatment compared to other diseases like COVID-19, the need to attend frequent clinic visits, and high disease prevalence in the area. For some people, high perceived severity stemmed from uncertainty about disease consequences, as they became aware of it only after the symptoms had worsened. Rarely, people recognised that delays in seeking care could lead to disfigurement, lesion spread, longer treatment, and delayed recovery. In contrast, others perceived CL as less severe (E5), due to low local incidence, awareness that it is a curable disease and not directly transmitted between individuals, and the belief that it causes less damage than other skin diseases like leprosy. However, even those with low perceived severity sometimes sought care out of general concern for their health. The combined effects of perceived risk of disease acquisition, severity, and disease awareness influenced whether individuals engaged in utilising biomedical healthcare or not.

### Perceived treatability

Individuals tend to seek timely healthcare if they believe in the availability of effective treatment for the disease. Receiving positive recommendations from healthcare professionals, family or friends, or witnessing others’ recovery from CL after biomedical treatment increased the perceived treatability among people (E6). However, subsequent treatment experiences of some CL patients during clinic visits shaped their perceptions regarding the disease’s treatability. Uncertainty regarding treatment efficacy, concerns about potential side effects, and prolonged or incomplete recovery, often shaped by hearing other patients’ stories at the clinic or, more rarely, through personal experiences, contributed to diminished perceived treatability, which may indirectly affect healthcare-seeking in the communities.

### Psychosocial impact on daily lives, associated costs and instrumental support

Our previous research on the psychosocial burden of CL revealed individuals’ negative experiences, including fear, disgust, body image concerns, negative societal reactions, treatment-related pain, anxiety from perceived disease severity, and emotional distress [4]. Building on this, we further analysed how these experiences influenced healthcare-seeking behaviour. Although some people perceived the lesions as a health threat, multiple factors contributed to delays in seeking medical care. Visible, large lesions, particularly on the face or hands, often trigger distress due to social attention, prompting some to seek treatment. However, delays were common due to fear of being labelled as a patient, the need for accompaniment to clinics (particularly for children and older adults), domestic responsibilities, and occupational constraints, such as farming or inability to work in mud with applied medications (E7). Anticipated costs, including medical expenses, transport, income loss, inability to obtain leave from the workplace, school absences, and upcoming examinations, further hindered timely care despite recognition of the lesion as a health threat.

During our creative community workshops, participants highlighted common challenges in accessing healthcare, with particular difficulties faced by older adults due to limited mobility and the need for caregiver support (E8). During creative activities, participants poignantly demonstrated how indirect costs due to productivity loss (i.e., loss of income generation) often outweighed health concerns. In one such performance, where participants took on the roles of doctor and patient, the doctor advised an older patient to rest for a couple of days after taking the medication. In response, the patient asked, ‘Will you feed my family while I rest?’, reflecting the tension between healthcare-seeking and sustaining daily livelihoods. This conflict often resulted in delayed or inadequate care utilisation. However, individuals who received support from family, friends, or community members were better able to overcome financial and logistical barriers and access timely biomedical treatment.

### Individual preference for different healthcare options

Personal choices for treatment options significantly influenced healthcare-seeking decisions, determining whether individuals pursued biomedical, Ayurvedic or traditional treatment, or home remedies. These choices were shaped by past experiences, perceived effectiveness, and accessibility of treatment. Some individuals preferred biomedical care due to positive outcomes from previous illnesses or observed recovery in other CL patients. Preferences also varied regarding the biomedical care, with individuals choosing between private and public healthcare facilities. Factors such as perceived procedural efficiency, shorter waiting times, and the personal belief that private healthcare providers offer higher-quality medicine and more personalised care influenced a preference for private medical centres (E9). Others favoured public hospitals for their free services and trust in government medical doctors. Those with greater trust in Ayurvedic medicine, particularly for skin conditions like pimples or chronic rashes, sought care from Ayurveda hospitals, viewing these treatments as effective and having minimal side effects due to natural ingredients. Some individuals opted for home remedies for the initial lesion, perceiving the condition as minor and manageable without medical intervention [43].

### Role of disease characteristics in CL healthcare-seeking

Specific characteristics of CL, including clinical manifestations, symptom progression and associated complications, failure of self-treatment, disease transmission, and prevalence, are important factors that directly or indirectly affect the perceived risk of disease acquisition, severity and awareness at individual and societal levels. In our previous work, we provided evidence around the patients’ perspectives on the insidious onset of CL, clinical manifestations, their interpretations of early symptoms and actions taken before seeking healthcare [43].

The initial presentation of CL lesions as small papules or nodules may lead to neglect, misinterpretation, or going unnoticed, hindering progression through certain landmarks of the healthcare-seeking pathway, such as symptom recognition and perceived health threats. If untreated, some lesions can progress to become large, ulcerated, painful, and persistent, with potential for secondary infections. These more severe manifestations often prompted individuals to seek care. For some, an open wound was perceived as the defining feature of CL, leading them to pursue biomedical treatment only once the condition had significantly worsened.

The small size of the sandfly, the insect vector of CL, further complicates the recognition of the initial lesion as CL. Although sandflies are abundant in certain environments, with which this rural population frequently interacts, people had not seen or lacked knowledge about the insect vector. Confusion over the vector recognition was common, with some misinterpreting sandflies as mosquitoes or sand fleas. Limited awareness of vector-borne transmission of CL and the link to sandflies prevented individuals from associating lesions with CL, delaying the perception of a health threat. Sandfly’s minuscule size also made it difficult for people to recognise a bite, often leading them to attribute lesions to more familiar insects. This contributed to delays in symptom recognition and threat perception. However, high prevalence and spatial clustering of CL cases within communities, particularly within households and neighbourhoods, fostered vigilance among people and prompted healthcare-seeking for suspicious lesions. Despite its prevalence in the community, varied clinical presentations of CL and the presence of other similar skin conditions (i.e., pimples, warts, oil bumps, mosquito bites) continued to challenge accurate recognition of CL at the community level.

In our earlier work, we demonstrated how people with CL in our study sites often attempted self-treatment using herbal remedies and over-the-counter medications [44]. However, in most cases, self-treatment provided only temporary symptomatic relief, addressing issues such as pain, itching, or swelling, rather than achieving complete lesion healing [44]. Over time, the lesion often reoccurred, worsened or failed to fully resolve. Therefore, failure of self-treatment was a decisive factor for some individuals to seek biomedical healthcare.

### Influence of social context on CL healthcare-seeking

The healthcare-seeking process for CL in rural Sri Lanka is shaped by factors embedded within the social context, which in turn influence individual-level factors and interpretations of disease characteristics. To better understand these socially rooted factors, we interpreted our findings based on the three sectors of health systems as defined by Kleinman [30]: popular, folk, and professional sectors.

### Popular sector

#### Family cohesion

Symptom recognition and subsequent decision-making were strongly influenced by collective experiences within families and communities, rather than by individual biomedical knowledge. People with CL commonly sought advice from ‘significant others’ within the popular sector, such as family members, neighbours, friends, and co-workers, to discuss symptoms and to explore possible actions and support. Our cross-sectional survey found that in most households (68.0%), women were the primary decision-makers regarding health matters [42]. These key decision-makers played an important role in determining when and where to seek treatment for ill family members. When women occupied multiple social roles, such as breadwinner, mother, and daughter, healthcare-seeking in CL became more difficult, despite their awareness of the health threat. However, receiving functional, verbal, and emotional support, such as help with transportation, childcare, household duties, farming, and accompaniment to health facilities, facilitated their burden alleviation and increased confidence in seeking care. Additionally, once family members assessed the symptoms and recognised the sick role, they often relieved affected individuals, particularly older adults, of household responsibilities.

#### Social networks and capital

Social networks and capital were instrumental in fostering collective awareness and promoting healthcare-seeking, as evidenced through participant observation and the diary study. Community interactions were crucial for disseminating health information. Informal gatherings near local boutiques, monthly meetings of community-based organisations (i.e., death benevolent societies, farmers’ and women’s societies), and discussions with key community figures like traditional healers and religious leaders were primary platforms for sharing information, including health messages (E10). Former CL patients also contributed by raising awareness, identifying lesions, and referring suspected cases to appropriate care (E11). Notably, even during the COVID-19 pandemic, community members and leaders remained engaged in identifying CL symptoms and encouraging healthcare-seeking. This proactive involvement, extending beyond information-sharing, significantly influenced healthcare-seeking behaviours, as consistently observed during participant observation and throughout the ECLIPSE project, highlighting the importance of social networks and capital in community health promotion.

#### Socially constructed misconceptions related to CL

Many misconceptions or myths related to CL prevailing in society, such as fears of CL causing internal organ damage, nervous system involvement, and the belief that sandflies lay eggs within the body, amplify perceptions of severity [4](E12), sometimes prompting earlier healthcare-seeking behaviour. Healthcare professionals acknowledged that some of these stories were created to explain the pathophysiology of CL and to encourage compliance and early healthcare-seeking. However, at times, these explanations had a negative impact, as they were exaggerated, perpetuated and contributed to unnecessary fear surrounding the disease.

#### General health-related behaviours and perceptions of community members

In our diary study, participants prioritised non-communicable diseases (NCDs) such as kidney disease, heart disease, hypertension, and cancer over CL as major health concerns in the area [55]. We observed a general lack of attention to health issues and cosmetic concerns, accompanied by poor healthcare-seeking behaviour (E13). Cultural perceptions influenced how symptoms were classified; some were viewed as ‘severe’ and requiring medical attention, while others were considered minor and manageable at home. Our data shows that healthcare-seeking was not limited to visiting hospitals for illnesses. Wound healing with herbal plants and indigenous medicine, and belief in supernatural and spiritual forces were culturally rooted. For illnesses perceived as ‘fatal’ or ‘severe’ (i.e., cancer, kidney disease), cultural practices like almsgiving and *Pirith* chanting (a Buddhist tradition of reciting protective verses) were commonly integrated with healing and wellbeing promotion. Most of these cultural practices were conducted alongside biomedical treatments and were not used as standalone approaches. In contrast, such practices were rarely applied to skin conditions. Instead, skin lesions were typically treated with herbal ointments or home remedies (E14), reflecting their perception as manageable and of low medical urgency. Occasionally, some individuals believed that home treatments might be ineffective if the condition resulted from a sandfly bite. On the other hand, some people attributed CL to supernatural forces, such as the ‘evil eye’, or viewed it as a consequence of ‘karma’, which can lead to inaction due to less locus of control.

### Folk and professional sectors

#### Knowledge, conduct and prioritisation of CL

Knowledge gaps surrounding CL persist not only among the general public but also within the folk and professional sectors in Sri Lanka. Interviews, CAGs, and CoP meetings revealed a limited understanding among traditional healers, who often regarded CL as another skin condition which required toxin removal from lesions (E15). In contrast, Ayurvedic doctors demonstrated sound knowledge of CL, including its clinical features, causation, and treatment. While Ayurvedic doctors claimed that they had effective treatment for CL in Ayurveda medicine, they typically referred suspected cases to public hospitals (E16). Biomedical doctors also showed a strong understanding of CL, primarily among those with clinical experience in endemic areas. However, some doctors still face challenges in making an accurate clinical diagnosis due to limited knowledge and experience with CL. Overcrowded healthcare settings further constrain doctor-patient communication, which is essential for addressing misconceptions, obtaining accurate patient histories, and supporting treatment adherence. Nevertheless, some doctors showed notable concern upon identifying CL lesions and referred patients for further diagnosis, even when the initial consultation was unrelated to CL.

Within the professional sector, CL remains a low priority in routine surveillance, particularly during public health emergencies (E17). This lack of prioritisation reflects a perception among healthcare professionals and authorities that CL imposes a relatively low psychosocial burden. Additionally, the absence of regular review meetings on CL at the regional level contributes to its limited attention. Some people with CL reported occasional visits from Public Health Inspectors (PHIs) during treatment, mainly for environmental assessments and prevention advice. Although PHIs play a key role in communicable disease control, community diaries and PERJs indicated that they were less integrated into community life than Public Health Midwives (PHMs), whose maternal and child health responsibilities promote closer community ties. This gap in community integration contributed to weaker communication between PHIs and the public, posing a barrier to effective health education and engagement.

### Structural determinants

Structural determinants play an indirect, less visible yet significant role in determining healthcare-seeking in CL. These determinants include health and non-health policies, health literacy, media influence, medical pluralism, and healthcare service-related factors such as availability, accessibility, acceptability, and quality. These are important in creating environments that either facilitate or hinder timely action by influencing both the individual and the broader social contexts of CL.

#### Health and non-health policies

Sri Lanka’s free healthcare policy enables universal access to CL treatment within the public healthcare sector, which supports early healthcare-seeking. However, policy-level gaps continue to undermine these efforts. As identified in our prior research on CL surveillance in Sri Lanka [56], structural weaknesses, such as underreporting and delayed case notification, contribute to the low perceived significance of CL, ultimately leading to delays in diagnosis and treatment. These surveillance deficiencies are further exacerbated by limited resource allocation, resulting in inadequate active case detection and reporting. Insights from interviews and CoPs meetings also highlighted operational challenges, including healthcare staff shortages, hospital overcrowding, and high workloads. These systemic issues divert attention and resources away from CL, reinforcing its low priority within the health system despite the availability of free treatment.

The healthcare professionals highlighted the lack of political and financial commitment as a major barrier to CL prevention. This includes insufficient support for public education on CL, training programmes for healthcare providers (E18) and investment in research on insect vector biology, parasites and their reservoir hosts, diagnostic tools and treatments (E19). Furthermore, our CoPs members identified the absence of updated guidelines informed by emerging evidence and new knowledge as a key contributor limiting effective clinical and public health responses to CL (E20). The COVID-19 pandemic and political-economic crises in Sri Lanka (2020–2022) worsened the situation, as health authorities needed to prioritise urgent issues, such as addressing maternal mortality and dengue outbreaks (E21).

Non-health policies, particularly in education and gender, indirectly shape CL healthcare-seeking by influencing people’s living standards, income, and decision-making power. For instance, Sri Lanka’s policy of free education has led to high literacy levels, reflected in our survey, where nearly 90% of participants had received secondary education [42]. While this creates a strong foundation for health literacy, awareness of CL remained low, likely due to the limited integration of CL-specific content in school curricula and public health messaging. Gender dynamics further intersect with these educational influences. As detailed earlier, females played a significant role in family health decision-making and active involvement in social networks, reflecting their agency and shared responsibility within families as an inherent aspect of the cultural context rather than as a reflection of gender stereotyping. (E22). Furthermore, stakeholders in the CoPs group repeatedly identified the absence of a people-centred approach and poor collaboration between health and non-health sectors as barriers limiting effective CL awareness and control.

#### Health literacy and media influence

The ability to comprehend health information, such as recognising early lesions as signs of CL and understanding it as a treatable condition, was another factor affecting early healthcare-seeking. We observed a strong community demand for accurate information about CL, with people actively seeking knowledge through mass and social media (E23). Yet, reliance on unofficial sources frequently led to misinformation and misconceptions, including exaggerated or inaccurate portrayals of the disease. During the interventional activities in the mother project, ECLIPSE, we found that using simple language and context-specific communication channels significantly enhanced public understanding and engagement with health information, irrespective of formal education levels.

#### Medical pluralism and healthcare service-related factors

Medical pluralism influenced healthcare-seeking behaviour for CL in Sri Lanka, with individuals navigating between self-treatment, traditional, Ayurvedic, and biomedical systems. These transitions, often driven by cultural acceptance, longstanding beliefs about skin conditions, perceived treatment effectiveness, community influence, and practical factors such as cost and distance, contributed to delays in accessing healthcare.

Availability of diagnostic and treatment centres for CL is limited in this rural CL-endemic setting. Our cross-sectional survey revealed that the percentage of people who had heard of CL was high in areas where a treatment centre was located in the periphery [42], while qualitative findings supported that proximity to treatment centres also contributed to shorter delays in seeking care. Yet, decentralisation of CL treatment centres remains a challenge due to the limited number of dermatologists in the country, logistical difficulties in drug storage and distribution, and the geographically scattered nature of CL cases. Dermatologists in our study also highlighted that the availability of CL treatment in peripheral hospitals (secondary level healthcare) is important (E24). However, treatment decentralisation challenges are not only health service-related but also linked to health policy decisions, particularly regarding resource allocation, workforce distribution, and integration of CL management into broader national healthcare planning.

Timely access to CL healthcare was often hindered by logistical challenges, including poor road conditions, limited public transport, and long distances to treatment centres, particularly for those in remote areas. Participants reported that inadequate transportation increased both travel time and costs, while safety concerns, such as wild elephant threats, further discouraged travel to hospitals. During the participant observation period, we witnessed that adverse weather conditions further restricted physical access to healthcare settings, particularly in remote agricultural areas (E25). While physical accessibility remained a challenge, the availability of free CL services through the public healthcare system provided a sense of financial comfort, supporting individuals in seeking care despite other logistical constraints (E26). The provision of separate clinic days for children and working individuals further improved access and convenience (E27). Apart from the deficits in availability and physical accessibility of CL-related health services, the acceptability of biomedical healthcare was generally high among the public. The quality of healthcare services was affected by concerns over the knowledge and competence of healthcare providers, the quality and side effects of the drugs provided, treatment procedures, as well as infrastructural limitations.

## Discussion

Based on our multi-method study, we developed an evidence-driven conceptual model that explains the complexity of healthcare-seeking in CL in rural Sri Lanka, laying a foundation for public health interventions to promote timely healthcare-seeking. Despite individual variation, we identified a common healthcare-seeking pathway with four landmarks: symptom recognition, perceived health threats, decisions on taking actions, and seeking help from the biomedical healthcare sector. Progression through these stages is shaped by the interaction of individual factors, disease characteristics, social context, and structural determinants. We have integrated the healthcare-seeking pathway with its influencing factors to develop our conceptual model. Grounded in the lived experiences of people with CL, communities, and healthcare providers, our conceptual model can be applied to similar resource-limited settings to understand and support early healthcare-seeking in CL.

Various theoretical frameworks explain health behaviours, including illness perception, risk-benefit assessment, and healthcare-seeking decisions [22, 26, 31]. However, as a skin-related NTD, CL poses unique challenges, including limited prevention measures, ambiguous symptoms, slow lesion progression, and use of multiple medical systems, particularly in rural contexts [57]. Existing models are inadequate to reflect these complexities and the broader sociopolitical and structural barriers that significantly shape healthcare-seeking and hinder NTD control. While Ramdas’s multidimensional model for health-seeking in CL [58] outlines a care-seeking pathway for CL, but it lacks clarity on how individual perceptions, social influences, and structural barriers interact. Our model addresses these gaps, integrating diverse micro-level constructs into four core landmarks and grouping determinants into four overarching domains.

In our model, individual context refers to personal or patient-level factors that directly influence healthcare-seeking in CL. However, these factors do not function in isolation; they are shaped by broader family, community, and societal influences, collectively forming the social context. Both individual and disease-related factors (proximal) are further mediated by cultural, social, and structural determinants (distal), which shape the socioeconomic position of the individual and contribute to health disparities and healthcare inequities [33]. The individual-level factors in our model, such as disease awareness [12, 59, 60], anticipated direct and indirect costs [61, 62], and perceived risk due to having CL patients in the same household and occupational exposures such as working in agricultural lands [61], align with global findings. Factors such as age [63, 64], gender [62, 63], income [63], ethnicity [64], and stigma [62, 65] have been identified in previous studies as influencing delays in healthcare-seeking, and our findings also suggest some of these factors play a role in the local context. Women, for instance, often carry the dual responsibilities of managing the household and caregiving while coordinating health-related decisions for family members [42]. Although this positioned women as active agents in family health decision-making, it also created competing demands on their time [44]. School children, on the other hand, depended on parents or guardians for treatment access and often faced interruptions in schooling. Older adults encountered challenges related to limited physical mobility and dependence on others for transport [44]. As the study was conducted exclusively within a Sinhala-majority community, the issue of ethnicity was not relevant and ethnic comparisons were not possible. Our findings suggest that these factors, while not directly determining delay, influenced healthcare-seeking in nuanced and context-dependent ways. Disease characteristics significantly affected healthcare-seeking [12, 62, 64]. Individuals were more likely to seek healthcare when symptoms appeared specific or disturbing, whereas early symptoms of CL resembling common conditions often led to delays in seeking treatment. This observation has important implications for improving healthcare-seeking. Diseases like CL, which are not life-threatening and initially present as minor, common skin lesions, require additional efforts from preventive healthcare services to effectively communicate the health threat and prompt timely healthcare-seeking. These efforts must be culturally competent and tailored to address key knowledge gaps within the specific context. Our model was developed through robust CEI, which brought together different types of knowledge. We incorporated Kleinman’s concept of medical systems as cultural systems [30], emphasising the influence of popular (i.e., family, community, and social networks), folk (i.e., traditional healers) and professional (i.e., Ayurvedic and biomedical healthcare professionals) on healthcare-seeking behaviours. Informed by wider debates on decolonisation and indigenous knowledge [66, 67], we recognise that these sectors operate within unequal relations of power and legitimacy. Hierarchies of knowledge are embedded in postcolonial societies such as Sri Lanka, and these societies often privilege biomedical knowledge as ‘scientific’ while positioning indigenous and experiential knowledge as less credible or valuable. Similar dynamics were evident in our study context: the professional sector, particularly the biomedical system, holds institutional and epistemic authority in CL case management. For instance, traditional practitioners we engaged with claimed that they could cure CL, yet such claims were generally disputed by biomedically trained clinicians whom we interviewed. Ayurvedic practitioners believed that treatments for CL were described in their classical texts but acknowledged the limitations of their approach in practice, frequently referring patients to biomedical facilities for diagnosis and treatment. The popular sector, consisting of families and community networks, functioned as the first actors in symptom interpretation and advice but remained largely unrecognised in biomedical health discourses. Although our ethnographic data demonstrate reliance on local knowledge, practices and traditions, we observed in all the patient journeys that people with CL ultimately relied on biomedical healthcare. We also acknowledge that our positionality as researchers may have shaped how these dynamics were interpreted [68], and we recognise the importance of continued reflexivity in understanding such social hierarchies and experiences.

Furthermore, our model acknowledges the importance of an individual’s immediate social environment in shaping symptom recognition, decision-making, and the utilisation of healthcare services. For example, while some studies report that women’s treatment delays in CL are due to social roles and family responsibilities [62], our findings show that familial and community support can facilitate timely care, despite these obligations. On the other hand, the priorities of community and health professionals often differed, with the community focused more on daily survival, while health professionals prioritised the health improvement of people. One key area for improvement is doctor-patient communication [69]. The model we presented here will address the balance we need between vertical and horizontal public health programs for leishmaniasis. Our model shifts emphasis from traditional vertical programmes in public health and highlights the importance of horizontal programmes, which embed contextual factors.

Despite systemic challenges in healthcare settings, such as staff shortages and high patient volumes, healthcare providers can still make a significant impact by encouraging CL patients to refer others with similar lesions and informing them that effective treatments are available. Similarly, traditional healers should be educated about CL and encouraged to refer patients for biomedical care, as Ayurvedic practitioners often do. Addressing broader social, cultural, and contextual determinants, strengthening both patient and community awareness is essential to promote early healthcare-seeking and reduce the public health burden of CL.

The influence of structural determinants on health is well established within the WHO SDH framework [33]. Our model highlights their subtle yet significant role in shaping healthcare-seeking decisions. In our analysis, we identified several factors that can be categorised as structural determinants, including medical pluralism. Medical pluralism is the coexistence of multiple therapeutic traditions within a given context [70]. While it reflects cultural richness, it can delay timely healthcare for CL. In countries like Ecuador [64], Suriname [71] and Ethiopia [60], people often rely on ethnomedicine (traditional medicine and practices) for CL. In our setting, although individuals initially turned to Ayurvedic and self-treatment, they ultimately accessed biomedical care. This underscores the need to integrate medical pluralism into health behaviour models and foster collaboration across healthcare systems to promote timely care. Drawing on the ECLIPSE experience, we argue that CL prevention and control must extend beyond the health sector. Policies in economics, politics, transportation, education, agriculture, and social services also shape healthcare-seeking. Availability of a strong public health system, with a network of primary healthcare workers extending to grassroots levels, alongside widespread mass media coverage and access to information in Sri Lanka, has the potential to improve health education and health literacy among the public further. However, current media and health communication strategies must move beyond top-down models, as culturally competent, bottom-up approaches could be more effective. Priorities include strengthening health infrastructure, increasing funding for NTD research and interventions, ensuring political and economic stability, and mitigating the effects of instability. Improving road infrastructure, expanding transport in remote areas, and tailoring health education to community needs are essential. Therefore, a multisectoral approach is imperative to improve healthcare-seeking in CL. Our model is grounded in empirical data generated through multiple methods, extensive fieldwork, and the CEI approach. The applicability of our model across contexts and health conditions is a main consideration. While the key landmarks along the healthcare-seeking pathway appear broadly universal, the determinants influencing progression through these landmarks are context-dependent, shaped by biological, social, cultural, and structural factors specific to each environment. For instance, symptom recognition may be a universal construct of healthcare-seeking, but its mechanisms and timing can vary with biological or environmental factors that influence the clinical presentation of CL across different settings [37, 72, 73]. Taking into account this contextual variability, the taxonomic elements of our model can be applied universally. The social and structural determinants that underpin progression along the health-seeking pathway will display substantial variation across contexts, both conceptually and empirically. Our model is adaptable to other diseases because it enables integration of context-specific variables such as disease characteristics, healthcare system dynamics, and sociocultural environments. These features support the utility of the model as a flexible and empirically grounded framework for examining healthcare-seeking behaviour in the context of NTDs as well as other health conditions.

## Conclusions

In conclusion, we have developed an evidence-based conceptual model of healthcare-seeking behaviour for CL based on a major, long-term mixed-methods study in rural Sri Lanka, integrating the main pathway that individuals follow when seeking healthcare for CL and key influencing factors. Our conceptual model can be further tested and applied in other endemic settings as it enables a context-specific and culturally appropriate understanding of the CL healthcare-seeking.

## Data Availability

All data generated or analysed during this study are included in this published article (and its supplementary information files).

## Abbreviations

CAG: Community advisory group
CEI: Community engagement and involvement
CL: Cutaneous leishmaniasis
CoP: Community of practice
DS: Divisional secretariat
ECLIPSE: Empowering people with Cutaneous Leishmaniasis: Intervention Programme to improve patient journey and reduce Stigma via community Education
HBM: Health belief model
NCDs: Non-communicable diseases
NTDs: Neglected tropical diseases
PERJ: Participant experience reflection journal
PHI: Public health inspector
PHM: Public health midwife
SDH: Social determinants of health
VL: Visceral leishmaniasis
